# Biologics for Targeting Inflammatory Cytokines, Clinical Uses, and Limitations

**DOI:** 10.1155/2016/9259646

**Published:** 2016-12-19

**Authors:** Peleg Rider, Yaron Carmi, Idan Cohen

**Affiliations:** ^1^The Department of Pathology, Sackler Faculty of Medicine, Tel-Aviv University, 6997801 Tel-Aviv, Israel; ^2^Galilee Medical Center, 22100 Nahariya, Israel

## Abstract

Proinflammatory cytokines are potent mediators of numerous biological processes and are tightly regulated in the body. Chronic uncontrolled levels of such cytokines can initiate and derive many pathologies, including incidences of autoimmunity and cancer. Therefore, therapies that regulate the activity of inflammatory cytokines, either by supplementation of anti-inflammatory recombinant cytokines or by neutralizing them by using blocking antibodies, have been extensively used over the past decades. Over the past few years, new innovative biological agents for blocking and regulating cytokine activities have emerged. Here, we review some of the most recent approaches of cytokine targeting, focusing on anti-TNF antibodies or recombinant TNF decoy receptor, recombinant IL-1 receptor antagonist (IL-1Ra) and anti-IL-1 antibodies, anti-IL-6 receptor antibodies, and TH17 targeting antibodies. We discuss their effects as biologic drugs, as evaluated in numerous clinical trials, and highlight their therapeutic potential as well as emphasize their inherent limitations and clinical risks. We suggest that while systemic blocking of proinflammatory cytokines using biological agents can ameliorate disease pathogenesis and progression, it may also abrogate the hosts defense against infections. Moreover, we outline the rational need to develop new therapies, which block inflammatory cytokines only at sites of inflammation, while enabling their function systemically.

## 1. Introduction

The use of recombinant proteins as biological drugs has been known for the past three decades; however, this field is continuously emerging and in the last decade an increasing number of new biologic entities (biologics) in the area of cytokines were developed. Biologics can be an antibody which neutralizes an inflammatory cytokine or blocks its receptors, decoy receptors targeting the cytokine, or a recombinant protein, which can either be receptor agonist or, alternatively, an antagonist that occupies and prevents receptor binding.

The benefits of cytokines as therapeutic targets are as follows: (i) unlike in chemical drugs, specific protein which mediate the inflammatory process can be inhibited; (ii) cytokines are well studied in animal models using neutralizing antibodies or genetic models like knockout mice; thus the process in which these cytokines are involved can be thoroughly researched; (iii) with the advancement of biotechnology techniques, the expression and isolation of highly purified recombinant proteins becomes a relatively easier and cheaper process than in the past years.

The drawbacks of cytokine therapy come due to the basic properties of cytokines: (i) cytokines are pleiotropic, meaning that they affect several processes in parallel; (ii) cytokines are also known to have redundancy, meaning that the effects achieved by blocking one specific cytokine activity can be compensated by others (although this can be also beneficial, since a biological agent can be replaced to different cytokine blocker when incomplete remission or in case of intolerance); (iii) the cytokine network is a regulated and balanced system and its alteration may lead to impaired immune response. For example, inhibiting proinflammatory cytokines can result in compromised host defense against infections. On the other hand, inhibition of regulatory cytokines can result in autoimmunity or tissue damage; (iv) the production and manufacturing of biologics is still an expensive process, since their production requires sterile conditions (i.e., GMP conditions) and multiple stages of purification; (v) compared to chemical drugs, recombinant cytokines and antibodies have limited shelf half-life, require special/controlled storage conditions, and are typically administrated by a physician.

In this review we discuss some of the key approaches of anticytokine blockers focusing on approved anti-inflammatory biologics. In particular, we highlight their beneficial effects and present their possible side effects and risk factors. Most importantly, we suggest several potential solutions for the anticytokine adverse effects and propose new approaches to this emerging field.

## 2. Therapeutic Use of Proinflammatory Cytokines

Cytokine therapy emerged from the need to increase immunity against tumors using the lymphocyte activator and proliferative factor, interleukin-2 (IL-2). Based on its remarked efficacy in mice, cancer patients bearing renal cell carcinoma (RCC) and melanoma were administered high doses of IL-2 in order to increase antitumor immunity [1, 2]. Unfortunately, systemic administration of IL-2 has been related to severe toxicity, mainly capillary leak syndrome, associated with edema and hypotension, damage to the kidneys, heart, and brain (as well as tachycardia, atrial fibrillation, fever and chills, muscle and joint pain, and catheter related urinary tract infections) [2, 3]. In spite of numerous restrictions and warnings, a recombinant modified version of IL-2 (aldesleukin) was approved in 1992 for metastatic RCC and in 1998 for metastatic melanoma patients [4].

As early as the 1990s, several years following the discovery of IL-1 by Auron et al. [5], IL-1 was used to treat cancer patients undergoing chemotherapy or patients suffering from anemia. It was assumed that since IL-1 has neutrophilic effects, it could restore neutrophil counts back to normal numbers in neutropenic patients [6–8]. However, IL-1 is a potent proinflammatory cytokine; thus the treatment resulted in toxicity with side effects such as fever, rigors, fatigue, joint aches, headache, and nausea.

Another example is IFN*α*, a cytokine involved in the response to viral infections. IFN*α* in a PEGylated form is given in order to increase antiviral immunity by elevated CD8+ cell response in cases of chronic hepatitis-B virus (HBV) and hepatitis-C virus (HCV) [9, 10] or in the case of immediate treatment for acute HCV. The IFN*α* can be given alone [11, 12] or together with the nucleoside analog, ribavirin [13]. This treatment facilitates the clearance of the HCV virus and can prevent the chronic disease which can result in cirrhosis and hepatocellular carcinoma [14]. However, this type I IFN cytokine can cause serious adverse effects that can result in limitation of the doses given or even in discontinuation of the treatment. Among these adverse effects are decreased granulocytes and thrombocytes production in the bone-marrow, flu-like symptoms, neuropsychiatric disorders, and autoimmunity syndromes, mainly thyroiditis [15].

## 3. Anti-Inflammatory Cytokine Biologics

### 3.1. Anti-TNF-*α* Biologics

TNF-*α* is a proinflammatory cytokine; it appears early during the response to trauma or bacterial infections and was first cloned in 1985 by several groups [16–19]. Initially it was described as a soluble factor with two important abilities, inducing hemorrhagic necrosis of tumors in vivo, combined with the ability to kill tumor cells in vitro [20]. TNF-*α* is a central alarm cytokine, which is mainly secreted from activated macrophages or dendritic cells in response to ligation of pattern-recognition receptors. Both TNF and IL-1 are attractive therapeutic targets, since they are the upstream factors of the inflammatory cascade. The role of TNF receptor signaling has been correlated with several diseases including rheumatoid arthritis (RA), Crohn's disease, atherosclerosis, psoriasis, sepsis, diabetes, and obesity [21]. TNF-*α* is expressed as a precursor, anchored to the cell membrane and further cleaved to its soluble form. TNF-*α* binds the inflammatory TNFR1 and regulatory TNFR2 and in addition to the inflammatory cascade affects cell death, proliferation, and differentiation [21].

The TNF-*α* inhibitor etanercept was the first biologic on the market for the treatment of RA. Etanercept is a FC fused recombinant form of a natural TNF inhibitor that was first described in 1988 [22] and later was found to be a soluble TNF receptor [23, 24]. Infliximab is a monoclonal chimeric human-mouse anti-TNF antibody and was approved by the FDA together with etanercept in 1998. Later on, by 2002, a fully human monoclonal antibody against TNF-*α* (adalimumab) was approved as well. Etanercept and anti-TNF antibodies carry differences in their abilities to bind TNF. While infliximab binds both monomeric and trimeric forms of TNF (the inactive and active forms), etanercept binds mainly the active trimeric form in a less stable manner [25], as well as binding TNF-*β* [26]. The anti-TNF antibodies are capable of lysing cells they bind by recruiting the complement system [27]. These differences appear in the molecules effectiveness against different inflammatory diseases and might be related to the antibodies binding to membranal TNF-*α* on T cells [28]. Infliximab was first approved for the treatment of severe Crohn's disease and later also for RA, where etanercept was first approved only for the treatment of RA. Infliximab was further approved for ulcerative colitis, psoriatic arthritis, ankylosing spondylitis, and chronic plaque psoriasis, and etanercept was further approved for psoriatic arthritis, ankylosing spondylitis, chronic plaque psoriasis, and juvenile idiopathic arthritis in children. Adalimumab is approved for rheumatoid arthritis, psoriatic arthritis, ankylosing spondylitis, Crohn's disease, ulcerative colitis, moderate-to-severe chronic psoriasis, moderate-to-severe hidradenitis suppurativa, juvenile idiopathic arthritis, and noninfectious uveitis. These days, certolizumab and golimumab are the newer, less studied anti-TNF-*α* antibodies most recently approved by the FDA for the treatment of RA, psoriatic arthritis, ankylosing spondylitis, Crohn's disease unresponsive to regular medications (certolizumab), and ulcerative colitis (golimumab).

Although the TNF inhibitors were shown effective for the treatment of skin and joint inflammation [29], they carry the risk of several adverse effects, mainly concerning infections. TNF-*α* is a fundamental factor for fighting intracellular bacteria and is therefore not surprising that TNF-*α* inhibition was shown to increase the risk for reactivation of tuberculosis [30]. In a 3-year French study of 69 newly diagnosed tuberculosis patients undergoing anti-TNF therapy, it was concluded that anti-TNF antibodies (infliximab and adalimumab) have a high risk for tuberculosis. Etanercept, the soluble TNF receptor, also carries such a risk but at a lower level [31]. It can be assumed that the differences in the anti-TNF strategies, which allow antibodies to be more effective against IBD, affect also the ability to inhibit the immune system to fight tuberculosis. Similarly, reactivation of HBV is higher during TNF inhibition [32]. Additionally, RA patients who were treated with anti-TNF antibodies experienced a higher rate of outbreaks of herpes zoster virus (HZV) compared to etanercept or disease-modifying antirheumatic drug (DMARD) treatments [33]. Blocking TNF plays an opposing role regarding the development of malignancies. On the one hand, TNF is an inflammatory mediator and the inflammatory process itself can lead to cancerous diseases [34]; hence, inhibiting TNF, like other proinflammatory molecules, can be beneficial in the aspect of cancer initiation and progression. On the other hand, TNF plays a role in cell proliferation, differentiation, and apoptosis [35], and, therefore, its inhibition can be a result and indeed was correlated with hematological malignancies, like increased hepatosplenic T cell lymphoma in young IBD patients treated with infliximab [36]. In addition, since TNF inhibitors are immunosuppressive drugs, they carry the risk for development of malignancies. Indeed, TNF inhibitors carry warnings for increased risk of hematological malignancies in children, adolescents, and young adults, primarily treated for ulcerative colitis or Crohn's disease also treated with immunosuppressant (azathioprine and/or mercaptopurine). The fact that TNF inhibitors are often combined with methotrexate which also increases the risk for malignancy [37] and, in addition, the association of diseases treated with TNF inhibitors, for example, IBD or RA, with increased risk for cancer [38–41] is making the direct link between TNF inhibitors and malignancies harder to determine. TNF inhibition, using infliximab or etanercept, was trialed for the treatment of congestive heart disease and not only were they shown to be inefficient but also increased the chance of hospitalization or death due to heart failure [42, 43]. Patients treated with anti-TNF therapy were also reported for increased risk for demyelinating disorders, like multiple sclerosis, optic neuritis, and acute transverse myelitis [44, 45]; paradoxical psoriasis consisting of severe skin lesions was observed in IBD patients treated with anti-TNF agents [46]. In addition, unlike anti-TNF antibodies, etanercept, which is not effective for the treatment of IBD, was correlated with the development of newly diagnosed ulcerative colitis and Crohn's disease in treated patients [47, 48].

### 3.2. Anti-IL-1 Therapy

Following the failure to use IL-1 as a therapeutic agent in order to treat neutropenic patients and the increasing data demonstrating the potency of this cytokine to induce inflammation, it was comprehended that IL-1 inhibition rather than IL-1 administration could be beneficial. Following inflammatory stimuli, like bacterial products, the proinflammatory cytokines, IL-1*α* and IL-1*β*, are elevated. However, an additional inhibitory protein that reduces these IL-1 molecules is secreted [49, 50]. The anti-inflammatory mediator was isolated in 1990, and the sequence of the IL-1 receptor antagonist (IL-1Ra) was published [51]. It is a cytokine, which belongs to the IL-1 family with about 40% similarity to IL-1*β* that binds the same IL-1 receptor type 1 (IL-1R1) albeit occupying it without inducing the signal transduction. The significance of the IL-1Ra as a natural anti-inflammatory cytokine is demonstrated by the genetic loss of function of the IL1RN gene. This results in a lethal systemic inflammatory disease with severe skin and bone involvement, termed deficiency of interleukin-1 receptor antagonist (DIRA) [52].

Anakinra is a recombinant nonglycosylated form of IL-1Ra that was approved in 2001 for the treatment of RA in adult patients that did not respond to other antirheumatoid drugs, like DMARD. Anakinra was shown beneficial for the treatment of RA by reducing symptoms and joint damage; however it is recommended to use when other biologics, like anti-IL-6 or anti-TNF therapies which are preferable, are refractory or contraindicated [53–55].

Anakinra competes with IL-1*β* for the receptor binding. The inflammasome-caspase-1 pathway mediates IL-1*β* activation and secretion. Mutations in the inflammasome related genes can result in autoinflammatory syndromes due to excess IL-1 [56]. Anakinra is therefore approved for the treatment of patients suffering from a form of Cryopyrin-Associated Periodic Syndromes (CAPS) called Neonatal-Onset Multisystem Inflammatory Disease (NOMID). CAPS is a common name for three autoinflammatory syndromes (familial cold autoinflammatory syndrome, Muckle-Wells syndrome, and NOMID), in which dysregulated inflammasome results in IL-1*β* activation and secretion and a broad inflammation occurs. Since IL-1 is the major mediator of these autoinflammatory diseases, it is obvious why anakinra, which blocks IL-1 activity, is preferable for therapy [57–60]. Anakinra is also given to other inflammatory or autoinflammatory diseases off-label. Familial Mediterranean Fever (FMF) is a hereditary chronic inflammatory disease which IL-1 plays a major role in, and blocking IL-1 reduces the symptoms [61, 62]. Anakinra was also shown to be effective in the case of nonhereditary chronic systemic inflammatory diseases like the adult-onset Still disease [63, 64], which involves arthritis, fever, and systemic inflammation or the childhood version—systemic-onset juvenile idiopathic arthritis (SJIA) [65–67]. In addition, there are more common inflammatory diseases like gout [68], hemodialysis patients [69], postmyocardial infarction cardiac remodeling [70], and type 2 diabetes, in which the glycaemia and beta-cell secretory function are improved [71], in addition to vast types of other inflammatory disorders responding to anakinra (reviewed n [72–75]).

Anakinra has a short half-life of about 6 h; treatment therefore requires frequent subcutaneous injections and the most common side effect of anakinra is injection site reaction. The short half-life of anakinra allows immediate withdrawal of the treatment if needed. During the administration of anakinra, the immune systems ability to fight infections is reduced. Meta-analysis of four RA trials using anakinra showed increased risk of infections, mainly pneumonia but also osteomyelitis, cellulitis, bursitis, herpes zoster, infected bunion, and gangrene [76]. Gouty arthritis patients treated with anakinra were also in increased risk for infections, mostly by* S. aureus* [68]. Since IL-1 is a neutrophil attractant and growth factor, the risk for neutropenia in patients treated with anakinra increased as well [77, 78], and during administration of anakinra neutrophil numbers must be followed. Anakinra is forbidden to patients receiving TNF blockers or patients getting live vaccines. The combination of anakinra together with corticosteroids or other immunosuppressive drugs increases the risk of infections. Combining anakinra with prednisolone was shown to risk RA patient with serious infections of* S. aureus*, hemolytic streptococci, and* E. coli* [79]. Patients with a history of tuberculosis are not recommended for anakinra treatment or for those participating in clinical trials, since the chance for reactivation of tuberculosis during administration of anakinra is high [76].

Rilonacept (also termed IL-1 trap), a dimer of IL-1R and IL-1R accessory protein (IL-1RacP) extracellular chains fused to the Fc fragments of IgG, was trialed and found effective for the treatment of CAPS [80]. Rilonacept was approved as biological drug in 2008, and canakinumab, a monoclonal anti-IL-1*β* antibody that was also shown beneficial for the treatment of CAPS [81–86], was approved in 2009. Like anakinra, both were shown to reduce symptoms in additional inflammatory diseases, such as gout [87, 88], and canakinumab was also shown to be effective for SJIA. Side effects associated with canakinumab resemble those of anakinra, such as increased risk of infections [89], neutropenia, and low platelet count [90]; therefore it is not recommended for patients with a high risk for infections. Canakinumab is administered once every four to eight weeks, dependent on disease severity, due to its extended half-life. Nonetheless, withdrawal will not terminate the effects of the drug immediately, like in the case of anakinra. Hyper-IgD syndrome (HIDS) is a genetic autoinflammatory syndrome associated with high IgD blood levels, caused by a mutation in the gene encoding mevalonate kinase (MK) [91]. TNFR1-associated periodic syndrome (TRAPS) is caused by intracellular accumulation of misfolded mutated TNFR1 and an elevated IL-1 production [92]. HIDS and TRAPS were shown to respond to anakinra [93–100]. Canakinumab is currently tested in a phase III trial in colchicine resistant FMF, HIDS/MK deficiency, and TRAPS patients (ClinicalTrials.gov identifier: NCT02059291). In addition, the effect of canakinumab on cardiovascular events and type 2 diabetes is currently held by the Canakinumab Anti-Inflammatory Thrombosis Outcome Study (CANTOS) trial [101].

### 3.3. Anti-IL-6

IL-6 is another major proinflammatory cytokine with pleotropic effects on the immune system. IL-6 is the ligand for IL-6 receptor (IL-6R). Following its binding, gp130, a transmembranal glycoprotein forms a homodimer and transmits the signaling. Unlike IL-1R1 or TNFR1, which are ubiquitous, IL-6R is restricted to hepatocytes, monocytes, macrophages, and lymphocytes. Another difference from the IL-1 and TNF cytokines is that the soluble form of IL-6R facilitates and induces the signal rather than serving as an inhibitor. Soluble IL-6R binds IL-6 and this complex further binds membranal gp130, which, unlike IL-6R, is expressed in all cell types. This kind of signaling is termed trans-signaling [102], a process which allows IL-6 to mediate its response on cells that lack IL-6R; among these are embryonic stem cells, endothelial cells, hematopoietic progenitor cells, osteoclasts, and neuronal cells [102]. The proinflammatory cytokines, IL-1 and TNF-*α*, were assumed to be responsible for the acute phase response of liver cells in vivo. Nevertheless, when hepatocytes response to stimulation by crude macrophage cytokines was compared to isolated cytokines IL-6, IL-1, and TNF, only IL-6 could induce fully comparable response [103]. Among the many IL-6 effects, it was found that it induces immunologic and metabolic responses. IL-6 can alter the T helper cell phenotype programming [104]; it can stimulate B cells, NK cells, osteoclasts, and cancer cells [105] and is secreted by a variety of cells; among these are lymphocytes, macrophages, endothelial cells, epithelial cells, and fibroblasts; these then play a major role in autoimmune diseases, especially RA, in which increased levels of IL-6 are found in synovial fluid [106]. The myeloma receptor antibody (MRA), a humanized antibody against IL-6R, was first trialed in 2003. It was then demonstrated to decrease serum acute phase protein in RA patients, which were not responsive to DMARD or other immunosuppressive drugs [107]. The MRA antibody was renamed tocilizumab and its efficiency for RA was demonstrated in a large trial consisting of 633 patients. The trial showed reduced disease activity [108] and the FDA approved tocilizumab in 2010 for the treatment of RA patients refractory to TNF inhibitors; additionally it was also shown efficient in another trial for the treatment of SJIA [109] where in 2011 the FDA expanded the use of the antibody to include the treatment of SJIA patients. Unfortunately, together with the benefits of IL-6 inhibition came adverse effects. Data pooled from five clinical trials, two ongoing extension trials, and one clinical pharmacology study summarized the following adverse effects among trial participants: serious infections mainly pneumonia, gastroenteritis, and urinary tract infections, opportunistic infections (such as tuberculosis, candidiasis), gastrointestinal perforation, and anaphylactic reactions. Other side effects were neutropenia and increased lipid levels, which are assumed to induce cardiovascular events [110]. Since IL-6 elevates CRP levels, its inhibition by tocilizumab results in milder elevation of CRP during infections. This can put the patients at risk since it is harder to diagnose an infection in patients undergoing treatment [111]. One of SJIA complications is Macrophage-Activating Syndrome (MAS), a life threatening disease, associated with impaired bone-marrow and liver functions. Tocilizumab treatment does not prevent or worsen MAS [112]; however, it does mask the clinical symptoms, again by reducing the CRP levels, which allow diagnosing the outbreak of this syndrome [113]. Blocking IL-1 with anakinra, on the other hand, was shown to reduce MAS severity in SJIA patients [114–116].

Siltuximab is a human-mouse chimeric anti-IL-6 antibody approved in 2014 for HIV-negative and herpes virus-8 negative patients for the treatment of multicentric Castleman's disease, a lymphoproliferative disorder associated with increased IL-6 in the enlarged hyperplastic lymph nodes [117]. Siltuximab was further studied for its beneficial anti-IL-6 effects in other malignancies, like multiple myeloma, myelodysplastic syndrome, prostate cancer, ovarian cancer and lung cancer, and cancer-associated cachexia and anorexia [118–122]. However, the treatment with siltuximab increases the risk of upper respiratory tract infections and other adverse effects including nausea, fatigue pruritus, increased weight gain, rash, hyperuricemia, thrombocytopenia, dyspnea, leukopenia, and neutropenia [123, 124].

### 3.4. Biologics Targeting TH17 Cytokines

Ustekinumab is a human monoclonal antibody against IL-12 and IL-23, which share the same IL-12p40 subunit. The antibody recognition of this cytokine reduces the differentiation of naïve CD4+ T helper cells into effector T cells, TH1, and TH17. Previously termed “IL-23-derived autoreactive CD4 T cells,” TH17 cells were named after IL-17 cytokine (which they produce) and are correlated with autoimmunity disorders including RA, lupus, colitis, and EAE [125, 126]. IL-12 and IL-23 and their associated T helper cells are correlated to psoriasis which is an immune-mediated chronic inflammatory skin disease, and psoriasis patients have an increased risk to develop psoriatic arthritis [127]. Ustekinumab was shown to be more effective compared to etanercept [128] and was approved in 2009 for plaque psoriasis and in 2013 for psoriatic arthritis. However, ustekinumab treated patients are recommended to receive prophylactic treatment due to increased risk of tuberculosis reactivation [129], as well as the issue of reduced CD4+ lymphocytes during this treatment, that should be taken into account [130, 131].

Secukinumab is a human anti-IL-17A antibody that was trialed and shown ineffective in clinical trials for the treatment of Crohn's disease, as the treatment aggravated the disease severity in addition to increased adverse effects, like upper respiratory tract infections and local fungal infections [132]. However, much like ustekinumab, IL-17 inhibition using secukinumab reduced symptoms and improved physiological functioning compared to placebo or etanercept in plaque psoriasis and was approved by the FDA in 2015. Secukinumab was also reported for its efficiency for psoriatic arthritis [133] and ankylosing spondylitis [134] and was approved for these indications. In March 2016 an additional monoclonal anti-IL-17 antibody—ixekizumab—was approved for patients with plaque psoriasis [135, 136]. Long-term data from experiences of these antibodies targeting effector helper T cells cytokines is required for further evaluation of the adverse effects and safety of these biologics.

## 4. Reducing Infections in Anti-Inflammatory Biologics

**Figure 1:**
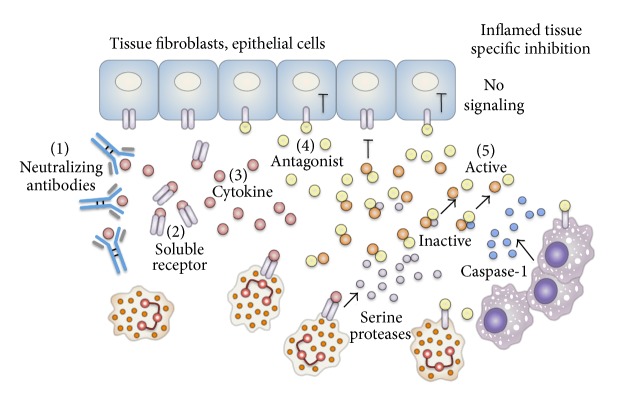
Biological drugs strategies for targeting inflammatory cytokines. The biologics can be composed of anticytokine or antireceptor neutralizing antibodies (1) or a soluble receptor that binds the cytokine (2). An inflammatory cytokine, like IL-1*β*, binds the IL-1R1 and the coreceptor IL-1R accessory protein (3) and transmits cell signaling, while an antagonist, like IL-1Ra, binds the receptor without recruiting the coreceptor (4), thus inhibiting signaling from the receptor and reducing the inflammation. Inflammation-dependent anticytokine strategy: enzymes such as neutrophil serine proteases or macrophage caspase-1 are released into the environment and cleave the two parts of the chimeric-IL-1Ra inactive precursor into an active antagonist (5), which blocks the receptors of tissue cells and the inflammatory cells.

Anticytokine therapy is a powerful tool to fight autoimmune and autoinflammatory diseases in addition to many other diseases in which the inflammatory process enhances the disease activity. For example, IL-1Ra, anakinra, was shown beneficial in vast types of diseases, among which are autoimmune RA [54], autoinflammatory diseases like CAPS [57–60], hereditary inflammatory FMF, improved beta cells function in type 2 diabetes [71], remodeling following myocardial infarction [70], smoldering myeloma [137], and a variety of other disorders [72]. Other inflammatory mediators like TNF-*α* and IL-6 have also great potential as targets in anti-inflammatory treatment. However, there is always a major cause for concern when systemically reducing inflammation by biologics that can compromise the patient ability to overcome infections. For example, the ability to reduce the rheumatoid process in patient's joints without inhibiting the neutrophils migration into the lungs in order to fight pneumonia is the objective for new biologics. One strategy to do so is to inject the patient with inactive biologic that would be activated whenever it meets the inflammatory site; the chimeric-IL-1Ra recently published carries such an approach [138]. This molecule is composed of the N-terminal peptide of IL-1*β* fused to IL-1Ra in its C-terminal side that mimics the structure of the precursor of IL-1*β*; thus it is expressed as an inactive procytokine. In the inflammatory sites, increased levels of neutrophil serine proteases (like elastase, cathepsin G, or chymotrypsin) [139], macrophages-derived PR3 and caspase-1 [140, 141], or granzymes from NK cells [142] are released from activated or dying cells and these enzymes cleave the N-terminal peptide of IL-1*β* and release the active free C-terminal cytokine part (Figure 1). For example in the inflamed joint of gouty arthritis patients, IL-1*β* is active due to the increased activity of neutrophils where the short-lived neutrophils are rich in serine proteases and are released to the site of inflammation [140]. As for the chimeric-IL-1Ra, the active IL-1Ra part is released in the same manner as IL-1*β* (i.e., an inactive precursor that transforms into an active cytokine due to the inflamed environment). At the same time, the patient's unaffected tissues are spared from the excessive systemic IL-1R1 blockade. Chronic inflammation in the microenvironment of tumors facilitates the tumors mechanisms of invasion and growth [143]. The tumor is surrounded with myeloid cells, rich with inflammatory enzymes and cytokines when IL-1 facilitates tumor growth, angiogenesis, and metastases [144, 145]. It was shown that the inflammatory tumorigenic microenvironment is derived from IL-1*β* secreted from the myeloid cells around the tumor, and the IL-1*α* secreted from the tumor cells accompanied with hypoxia, necrosis, or DNA damage [146–149]. It was therefore why anti-IL-1 therapy was suggested for trials in cancer patients [150, 151]. Recently, it was shown that IL-1*α* neutralization using a monoclonal antibody would be beneficial in cancer patients in prolonging their survival [152, 153]. Cancer patients are often treated with immunosuppressive and bone-marrow suppressing drugs; therefore, they are exposed to increased risk of infections. Thus, biologics like the chimeric-IL-1Ra that might reduce the inflammatory process in the tumor site without reducing the patient's ability to fight infection is a desirable approach. In order to inhibit cell surface TNF, in a cell-type restricted manner, Efimov et al. constructed bispecific antibody that recognizes both the F4/80 macrophage marker and the membranal TNF-*α* [154]. In this manner, the antibody favors binding of TNF-*α* on myeloid cells rather than free TNF-*α* or T lymphocytes derived TNF-*α*. The aim was to reduce anti-TNF side effects by blocking macrophage-derived inflammation, while maintaining T cell activity. The authors claim that this antibody can prevent reactivation of latent tuberculosis and reduce anti-TNF liver toxicity.

## 5. Concluding Remarks

Unregulated levels of cytokines are central mediators of many inflammatory diseases. Targeting these cytokines using recombinant anti-inflammatory cytokines, recombinant soluble receptors, or antibodies against cytokines has demonstrated preferable clinical outcomes in patients with autoimmune diseases, which are refractory to glucocorticoids treatments. However, systemic cytokine blocking suffers from a number of serious limitations. For one, the lack of danger signals, which is crucial for adequate immune cell activation as well as hematopoiesis alterations, common features in all biologics, expose the host to increased risks of infections. In addition, the pleiotropic nature of most cytokines and their necessity to the function of multiple cell types across different organs make it almost impossible to inhibit their signaling cascade in a long-term therapy without severe complications. Therefore, new approaches based on site-restricted biologics, which maintain the cytokine activity in other sites, are highly advised.
